# A binary logistic regression model with complex sampling design of unmet need for family planning among all women aged (15–49) in Ethiopia

**DOI:** 10.4314/ahs.v17i3.6

**Published:** 2017-09

**Authors:** Demeke Lakew Workie, Dereje Tesfaye Zike, Haile Mekonnen Fenta, Mulusew Admasu Mekonnen

**Affiliations:** Department of Statistics, Bahir Dar University, Bahir Dar, Ethiopia

**Keywords:** Complex sampling design, Ethiopia, family planning, Performance Monitoring and Accountability, unmet need

## Abstract

**Background:**

Unintended pregnancy related to unmet need is a worldwide problem that affects societies. The main objective of this study was to identify the prevalence and determinants of unmet need for family planning among women aged (15–49) in Ethiopia.

**Methods:**

The Performance Monitoring and Accountability2020/Ethiopia was conducted in April 2016 at round-4 from 7494 women with two-stage-stratified sampling. Bi-variable and multi-variable binary logistic regression model with complex sampling design was fitted.

**Results:**

The prevalence of unmet-need for family planning was 16.2% in Ethiopia. Women between the age range of 15–24 years were 2.266 times more likely to have unmet need family planning compared to above 35 years. Women who were currently married were about 8 times more likely to have unmet need family planning compared to never married women. Women who had no under-five child were 0.125 times less likely to have unmet need family planning compared to those who had more than two-under-5.

**Conclusion:**

The key determinants of unmet need family planning in Ethiopia were residence, age, marital-status, education, household members, birth-events and number of under-5 children. Thus the Government of Ethiopia would take immediate steps to address the causes of high unmet need for family planning among women.

## Background

Family planning reduces poverty, maternal and child mortality; empowers women by lightening the burden of excessive child bearing and it reduces environmental degradation by stabilizing the population of the planet^1^–^3^. Millions of women worldwide would prefer to avoid becoming pregnant either right away or never get pregnant, but are not using any contraception^4^,^5^. Unintended pregnancy related to unmet need is a worldwide problem that affects women and their families and societies at large^2^,^6^. The concept of unmet need indicates the gap between some women's reproductive intentions and their contraceptive behavior for Family Planning (FP)^3^,^4^,^7^. Serving all women in low income countries that currently have an unmet need for modern methods would prevent an additional 54 million unintended pregnancies, including 21 million unplanned births, 26 million abortions (of which 16 million would be unsafe) and seven million miscarriages; this would also prevent 79,000 maternal deaths and 1.1 million infant deaths^8^.

Contraception and unmet need levels varied across countries of the world, the lowest contraception and highest unmet need, being in sub-Saharan Africa; 25 % of women of reproductive age who are married or in union have an unmet need for family planning^9^,^10^. According to Demographic and Health Survey^7^ currently married women unmet need for FP was 22% for Tanzania and 10% for Zimbabwe. According to the 2000, 2005 and 2011 Ethiopian demographic and health survey, the level of unmet need for women who are married or in union in Ethiopia was 37, 36 and 25.3% respectively. The PMA 2020/Ethiopia survey in round 2 and 3 were 16.2 and 16.5% of all women in the age group of 15–49 had an unmet need for FP^11^. In a study conducted in Harare, 33.3 % pregnant women reported that their most recent pregnancies were unintended of which, 50 % had unintended child births and the other 50 % ended with induced abortion^12^. A study conducted in Butajira on determinants of low family planning use showed that unmet need of contraception was 52.4% of which 74.8% was attributed to spacing and the rest for limiting^8^. A study conducted in Misha-Southern Ethiopia and Dangila-Amhara region revealed that 26.5%^13^ and 17.4 %^2^ of married women had unmet need for family planning.

In these studies, no one took into account complex sampling design and fitted the data using binary logistic regression even if this data is from multistage sampling. Therefore fitting such data without considering the survey sampling design may lead to biased estimates of parameters and incorrect variance estimate^14^. For this reason, the current study used the PMA2020/Ethiopia survey data to account for complex sampling design to find the prevalence and key determinants of unmet need for FP among all women aged (15–49) in Ethiopia using binary logistic regression model with complex sampling design.

## Methods

### Sampling design, sample size and data course

The PMA 2020/Ethiopia was conducted in April 2016 at round-4 from 7494 women with two stage-stratified sampling. A sample of 221 enumeration areas (EAs) was drawn by the Central Statistical Agency from its master sampling frame. Each EA was listed and mapped; 35 households were randomly selected. Occupants in selected households were enumerated and eligible women of reproductive age (15–49) were contacted and consented for interview. Data collection was conducted between March and April, 2016 and 7494 women considered in this study. The data collection was led by the Addis Ababa University's School of Public Health at the College of Health Sciences (AAU/SPH/CHS), in collaboration with regional universities, the Federal Ministry of Health and the Central Statistics Agency. Overall direction and support was provided by the Bill & Melinda Gates Institute for Population and Reproductive Health at the Johns Hopkins Bloomberg School of Public Health. The household and female surveys were carried out by resident enumerators (REs) trained to use smart phones. REs are typically women over the age of 21 and hold a high school diploma or higher level of educational attainment^15^.

### Statistical analysis

Unmet contraceptive need of women defined as women are considered to have an unmet need for spacing or limiting if they are: at risk of becoming pregnant, not using contraception, and either do not want to become pregnant within the next 2 years or are unsure if or when they want to become pregnant or pregnant with a mistimed pregnancy or postpartum amenorrhoeic for up to 2 years following a mistimed birth and not using contraception^3^,^4^,^8^,^16^. Thus, unmet need for family planning was the study variable and coded 1 for unmet need and 0 for no unmet need.

Data was entered into STATA-12 and analyzed using SPSS-21. Binary logistic regression with complex sampling design was fitted for the unmet need outcomes. Married women are disaggregated by various background characteristics to have an insight of their characteristics.

All background characteristics of women used in this study were categorical though the dependent variable, unmet need was binary in nature. Unmet need of contraception with 95% confidence interval was computed. Bi-variable and multi-variable logistic regression model with complex sampling design were used to identify prevalence and the key determinants of unmet need for FP among all women aged (15–49) in Ethiopia. The binary logistic regression considers that the data are collected using simple random sampling where each sampling unit has the same probability of being chosen from the population. However, if the data is collected using complex survey sampling designs, binary logistic regression may lead to biased estimates of parameters and incorrect variance estimates. This is because, including sampling design when the data was obtained from complex sampling design is better in order to make statistically valid inferences from the finite population^14^,^17^. Only co-variates that were statistically significant at the bi-variable level were included in the multi-variable binary logistic regression to control for confounding. Though many variables were considered in the analysis, only covariates significantly associated with contraception were reported.

## Results

### Socio-demographic characteristics of all women aged (15–49) in Ethiopia

Table 1 shows socio-demographic characteristics of 7494 women of reproductive age (15–49) who were recruited for the survey. More than seventy five percent of the respondents were from the rural areas and the majority was in the age group of 15–24 years which contributed 3079 (41.1%) followed by 25–34 years. Moreover, 3118 (41.6%) of women were illiterate and 2885 (38.5%) had had primary education. About 4671 (62.3%) of women were currently married and 613 (12.9%) of them reported that their partner had other wives. Of the study participants, 3242 (43.3%) had no livestock while 4572 (61.0%) had 3–6 members in the house.

**Table 1:** Socio-demographic characteristics among women aged (15–49) in Ethiopia, March to April, 2016

Variables		Frequency	Percent
	15–24	3079	41.1
Age (5-year groups)	25–34	2420	32.3
	35+	1995	26.6
Place of residence	Urban	1803	24.1
	Rural	5691	75.9
	Never	3118	41.6
	Primary	2885	38.5
Highest level of school attended	Secondary	1168	15.6
	Technical	202	2.7
	Higher	116	1.5
	Currently married	4671	62.3
	Currently living with man	112	6.9
Marital status	Divorced or separated	515	6.9
	Widow	200	2.7
	Never married	1994	26.6
	Poor	2893	38.6
Wealth quintile	Middle	1440	19.2
	Rich	3161	42.2
Number of household members in the roster	1–2	743	9.9
	3–6	4572	61.0
	7+	2179	29.1
Married once or more than once	once	4508	82.0
	More than once	989	18.0
Partner has other wives	No	4147	87.1
	Yes	613	12.9
Livestock on homestead	No	3242	43.3
	Yes	4252	56.7
Own livestock	No	1882	25.1
	Yes	5603	74.9
Last baby still alive	No	178	3.5
	Yes	4853	96.5

### Prevalence and reason of unmet need for FP among all women aged (15–49) in Ethiopia

Table 2 shows various responses by women of reproductive age (15–49) towards the usage of family planning services. The prevalence of unmet need for FP was 1217 (16.2%) at the time of interview in the study area of Ethiopia. The unmet need for spacing and limiting of births was found to be 768 (10.2) and 449 (6.0%) respectively while 2382 (31.8%) of women were not sexually active. The major reason for not using FP among women of reproductive age group was ‘not married’, not having sex, menopausal/hysterectomy, sub/in fecund, no menses since last birth, breastfeeding and husband away 2821(72.8%); 440 (14.5%) were fatalistic; some had religious prohibition; 419 (13.9%) were afraid of the side effects and health concerns and 304 (10.1%) had opposition from the partner.

**Table 2:** Distribution of type of unmet need for FP among women aged (15–49) in Ethiopia, March to April, 2016

		Frequency	Percent
Unmet need	Missing	4	0.1
	Not sexually active	2382	31.8
	Unmet need for spacing	768	10.2
	Unmet need for limiting	449	6.0
	Using for spacing	1317	17.6
	Using for limiting	769	10.3
	No unmet need	1085	14.5
	In fecund or menopausal	719	9.6
**Prevalence of unmet need of FP (7,494)**			
Total unmet need	no unmet need	6277	83.8
	unmet need (spacing and/or limiting)	1217	16.2
	No Need^a^	2821	72.8
	up to god/fatalistic or religious prohibition	440	14.5
	Oposed^b^	304	10.1
	knows no method and Source	81	2.7
Reason not using FP*:	fear of side effects or health concerns	419	13.9
	lack of access/too far or costs too much	35	1.2
	preferred method not available or no method available	29	0.9
	inconvenient to use	29	0.9
	other	545	18.0

* multiple responses.

a not married, not having sex, menopausal/hysterectomy, sub/infecund, no menses since last birth, breastfeeding and husband away. Among this all the majority 1714(36.2%) was not married.

b opposed by partner, respondent, others or interferes with other body.

### Family planning methods used and exposure among all women aged (15–49) in Ethiopia

Table 3 revealed that various responses by women of reproductive age (15–49) towards the most effective current FP methods. Among all women of reproductive age, only 1343 (17.9%) and 1246 (35.3%) reported that they were visited by a health worker and talked to about FP in last 12 months respectively. In this study, 5062 (67.8 %) women reported that they had heard information about family planning from different sources within the last 12 months. The most frequently used source of information for family planning was radio 2655 (52.4%), television 1836 (36.3%) and newspaper 571 (11.3%). In this study, 4416 (81.7%) of women knew a place to obtain a method of FP and 33.4% of women were using most effective current FP method. The favorite modern contraceptive ever practiced among women of reproductive age was injectable that is 1254 (60.4%) and the least was beads which constituted 3 (0.1%). Among FP users, 1160 (48.5%) of women obtained FP from governmental health center; and 738 (30.9%) obtained FP were from a governmental health post when they started.

**Table 3:** Most effective current FP method among women aged (15–49) in Ethiopia, March to April, 2016

	Variables	Frequency	Percent
Most effective current FP method	Female sterilization	15	0.7
	Implants	477	23.0
	IUD	42	2.0
	Injectables	1254	60.4
	Pill	143	6.9
	Emergency	11	0.5
	Male condoms	27	1.3
	Beads	3	0.1
	LAM	12	0.6
	Rhythm	78	3.8
	Withdrawal	13	0.6
	Other traditional	2	0.1
Do you know a place you can obtain a method of FP	No	992	18.3
	Yes	4416	81.7
Did you obtain the method you wanted?	No	187	7.5
	Yes	2304	92.5
	Governmental hospital	98	4.1
	Governmental health center	1160	48.5
	Governmental health post	738	30.9
	NGO	17	0.7
Where did you obtain method when you started using	Volunteer	2	0.1
	FP clinic	32	1.3
	Private hospital	223	9.3
	Pharmacy	94	3.9
	Shop	3	0.1
	Friend relative	12	0.5
	Other	11	0.5
Visited by health worker about FP last 12 months	No	6139	82.1
	Yes	1343	17.9
Visited health facility in last 12 months	No	3961	52.9
	Yes	3527	47.1
Talked to about FP at health facility	No	2280	64.7
	Yes	1246	35.3
Exposure to Media*	Radio	2655	52.4
	Television	1836	36.3
	Newspaper	571	11.3

* multiple responses.

Among women who participated in this study, the smallest unmet need was from Addis Ababa 12(3.2%) followed by Dire Dawa 2(8%) and Amhara 157 (9.2%) regions whereas women who had highest unmet need were from Benishangul Gumuz 28(26.4%), Oromia 644(22.4%) and Harari 4(18.2%). The prevalence of unmet need for FP in SNNPR 1681(16.2%) was similar with the national results (Table 4).

**Table 4:** Total unmet need for FP by region in Ethiopia, 2016

		Total unmet need	
		
		No unmet	Unmet
Tigray	Count (%)	429 (86.0)	70 (14.0)
Afar	Count (%)	78 (84.8)	14 (15.2)
Amhara	Count (%)	1544 (90.8)	157 (9.2)
Oromiya	Count (%)	2233 (77.6)	644 (22.4)
Ethiopia Somali	Count (%)	67 (89.3)	8 (10.7)
Benishangul Gumuz	Count (%)	78 (73.6)	28 (26.4)
SNNPR*	Count (%)	1409 (83.8)	272 (16.2)
Gambella	Count (%)	32 (86.5)	5 (13.5)
Harari	Count (%)	18 (81.8)	4 (18.2)
Addis Ababa	Count (%)	366 (96.8)	12 (3.2)
Dire Dawa	Count (%)	23 (92.0)	2 (8.0)
Total	Count (%)	6277 (83.8)	1216 (16.2)

* south nations and nationalities of peoples of Representative

### Factors associated with unmet need for FP among all women aged (15–49) in Ethiopia

Table 5 shows the bi-variable and multi-variable factors associated with unmet need for FP among women of reproductive age group in Ethiopia. After controlling for the possible confounders using forward stepwise likelihood ratio method, age, marital status, number of household members, birth events, number of children under five, place of residence, highest level of school attended and knew a place of FP method were found as significantly associated factors for the unmet need for FP.

**Table 5:** Factors associated with unmet need for FP among aged (15–49) in Ethiopia, March to April, 2016

Variables		Total Unmet Need		COR (95% CI)	AOR (95% CI)	Over all P-value

		No	Yes n (%)			
Place of Residence	Urban	1672	130(7.2)	0.33 (0.273, 0.399)*	0.717 (0.556, 0.924)*	<0.0001
	Rural	4605	1087(19.1)	1	1	
Age in groups	15–24	2766	313(10.2)	0.466 (0.397, 0.547)*	2.266 (1.644, 3.123)*	<0.0001
	25–34	1906	514(21.2)	1.112 (0.959, 1.288)	1.237 (0.989, 1.547)	
	35+	1605	390(19.5)	1	1	
Marital Status	Currently married	3549	1121(24.0)	19.5 (13.648, 27.863)*	8.077 (5.21, 12.523)*	<0.0001
	Living with man	87	25(22.3)	17.4 (9.857, 30.718)*	7.344 (3.654. 14.761*	
	Divorced/separated	488	28(5.4)	3.492 (2.077, 5.87)*	1.498 (0.825, 2.722)	
	Widow	189	12(6.0)	3.785 (1.900, 7.542)*	1.434 (0.663, 3.103)	
Never married	1962	32(1.6)	1	1		
	Never	2418	701(22.5)	4.816 (2.182, 10.628)*	0.323 (0.186, 0.559)*	
highest level of school attended	Primary	2479	406(14.1)	2.724 (1.231, 6.026)*	0.417 (0.240, 0.726)*	<0.0001
	Secondary	1076	93(8.0)	1.434 (0.635, 3.241)	0.499 (0.278, 0.896)*	
	Technical	191	10(5.0)	0.907 (0.332, 2.476)	0.460 (0.184, 1.150)	
	Higher	109	7(6.0)	1	1	
Number of household members	1–2	686	56(7.5)	0.279 (0.209, 0.373)*	1.506 (1.011, 2.244)*	<0.0001
	3–6	3908	665(14.5)	0.578 (0.507, 0.658)*	0.763 (0.627, 0.928)*	
	7+	1683	496(22.8)	1	1	
	No birth	2394	67(2.7)	0.063 (0.048, 0.082)*	0.162 (0.098, 0.268)*	
birth events	1–2	1476	308(17.3)	0.470(0.398, 0.554)*	0.346 (0.251, 0.476)*	<0.0001
	3–5	1411	399(22.0)	0.637 (0.544, 0.746)*	0.528 (0.415, 0.670)*	
	6+	996	442(30.7)	1	1	
number of children under 5	No under 5	3797	201(5.0)	0.044 (0.030, 0.066)*	0.125 (0.079, 0.199)*	<0.0001
	1 – 2 under 5	2420	953(28.3)	0.331 (0.227, 0.484)*	0.654 (0.426, 1.003)	
	> 2 under 5	51	61(54.5)	1	1	
Knew a place obtain a method of FP	No	851	141(14.2)	0.513 (0.423, 0.621)*	0.590(0.467, 0.744)*	<0.0001
	Yes	3340	1076(24.4)	1	1	

**Keys:** COR (Crude odds ratio); AOR (Adjusted Odds Ratio); CI (Confidence Interval);

1 (Reference Category);

* Odds Ratio is a significant at α=0.05

## Discussion

This paper was conducted to investigate the magnitude and determinants of unmet need for FP among all women of reproductive age group. In this paper, we also aimed at finding out the determinants of unmet need for FP in the community. Of the 7494 women of reproductive age group, 1217 (16.2%) had an unmet need for FP 10.2% for spacing and 6% for limiting. There is a clear relationship between women's age and the level of unmet need. Unmet need is typically low in women of age 15–24 and peaks for many women in their thirties and then declines in the forties. It is perhaps the experience and awareness about contraception among older women that reduces the likelihood of unmet need among them.

In addition, the country's five-year Growth and Transformation Plan (GTP) works to gender equality empower all women and girls and eliminate early marriage^18^. The level of unmet need for FP in this study (16.2 %) is lower than the study conducted on currently married women at Dangila (17.4%)^9^ but seemed steady with the PMA 2020/Ethiopia survey of all women of reproductive age group in round 2 and 3 (16.2 and 16.5%)^11^. This may need more commitment of the Ethiopian government and NGO to achieve a 3.7% unmet need for FP by 2030^18^.

Relatively lower unmet need among urban women in Ethiopia could be ascribed to accessibility of health services and awareness of the urbanites on family planning methods. Better mass media coverage in urban compared to rural areas might have increased knowledge and practice. Urban women could also be more enlightened and autonomous to utilize health services compared to their counterparts in rural areas^9^,^19^.

Women's educational status is positively associated with contraceptive prevalence in Ethiopia. Studies elsewhere revealed a similar pattern of relationship between educational status and maternal health service including family planning utilization^9^,^20^. Highest level of school attended could probably give women better chance to understand uses of contraception to reduce fertility, maternal and child morbidity and mortality. Women who attended higher level of school could avoid the negative effects of family planning methods by reading different publications about it and getting appropriate advice from a service provider thereby increased their consistent use.

Number of birth events among women is also positively associated with contraceptive prevalence in Ethiopia. As the number of events increased there is less unmet need for FP in Ethiopia. Meanwhile, women who had no under-five child were less likely to have unmet need for FP compared to those who had more than two under five. This finding was in line with a study conducted in Mojo town, Enemay, East Gojjam Zone and Bihar, India^3^,^9^, ^21^,^22^. Women who had more births avoided the negative effects of family planning methods by reading different publications about the side effect of contraception methods and getting appropriate advice from a service provider thereby increased their consistent use.

## Conclusion

This study was aimed to make a contribution to identify the prevalence and key determinants of unmet need by using a binary logistic regression model with complex sampling design to account for the complexity of the survey. The key determinants of unmet need for FP in Ethiopia were place of residence, age of women, marital status, highest level of school attended, number of household members, birth events, number of children under five and knew a place from which to obtain a method of FP. The influence of these factors can be used to develop the strategies of reducing unmet need FP in Ethiopia.

The unmet need in Ethiopia is still very high, especially in currently married women. A high rate of unmet need for modern contraceptive methods might potentially lead to increased rates of unwanted pregnancies and induced abortions. Thus the Government of Ethiopia will have to take immediate steps to address the causes of high unmet need for family planning among women.

## List of Abbreviations

AAU: Addis Ababa University
AOR: Adjusted Odds Ratio
CHS: College of Health Sciences
CI: Confidence Interval
COR: Crude Odds Ratio
EDHS: Ethiopia Demographics Health Survey
ESA: Ethiopian Statistical Agency
FP: Family Planning
GTP: Growth and Transformation Plan
LAM: Lactational Amenorrhea Method
OR: odds Ratio
PMA2020: Performance Monitoring and Accountability 2020
REs: Resident Enumerators
SAS: Statistical Analysis System
SD: standard Deviation
SPH: School of Public Health
SPSS: statistical packages for social Sciences

## Availability of data and materials

The dataset supporting the conclusions of this article is held by PMA2020/Ethiopia Survey Project and the de-identified data may be made available based on written request and approval by PMA 2020's coordinating center in Baltimore.
